# Potential Prognostic Role of ^18^F-FDG PET/CT in Invasive Epithelial Ovarian Cancer Relapse. A Preliminary Study

**DOI:** 10.3390/cancers11050713

**Published:** 2019-05-23

**Authors:** Anna Myriam Perrone, Giulia Dondi, Giacomo Maria Lima, Paolo Castellucci, Marco Tesei, Sara Coluccelli, Giuseppe Gasparre, Anna Maria Porcelli, Cristina Nanni, Stefano Fanti, Pierandrea De Iaco

**Affiliations:** 1 Department of Medical and Surgical Sciences (DIMEC), Unit of Gynecologic Oncology, University of Bologna, 40138, Bologna, Italy; giulia.dondi@gmail.com (G.D.); marco.tesei2@gmail.com (M.T.); sara.coluccelli2@unibo.it (S.C.); giuseppe.gasparre@gmail.com (G.G.); pierandrea.deiaco@unibo.it (P.D.I.); 2 Nuclear Medicine Unit, University of Bologna, S. Orsola-Malpighi Hospital Bologna, University of Bologna, 40138 Bologna, Italy; giacomo.maria.lima@gmail.com (G.M.L.); paolo.castellucci@aosp.bo.it (P.C.); cristina.nanni@aosp.bo.it (C.N.); stefano.fanti@aosp.bo.it (S.F.); 3 Center for Applied Biomedical Research (CRBA), University of Bologna-S. Orsola Hospital, 40138 Bologna, Italy; 4 Department of Pharmacy and Biotechnology (FABIT), University of Bologna, 40126 Bologna, Italy; annamaria.porcelli@unibo.it

**Keywords:** ovarian cancer, PET/CT, relapse, SUV_max_, targeted therapy, prognosis

## Abstract

Epithelial ovarian cancer (EOC) is the most lethal gynecological malignancy, with relapse occurring in about 70% of advanced cases with poor prognosis. Fluorine-18-2-fluoro-2-deoxy-d-glucose PET/CT (^18^F-FDGPET/CT) is the most specific radiological imaging used to assess recurrence. Some intensity-based and volume-based PET parameters, maximum standardized uptake values (SUV_max_), metabolic tumor volume (MTV) and total lesion glycolysis (TLG), are indicated to have a correlation with treatment response. The aim of our study is to correlate these parameters with post relapse survival (PRS) and overall survival (OS) in Epithelial Ovarian Cancer (EOC) relapse. The study included 50 patients affected by EOC relapse who underwent ^18^F-FDGPET/CT before surgery. All imaging was reviewed and SUV_max_, MTV and TLG were calculated and correlated to PRS and OS. PRS and OS were obtained from the first relapse and from the first diagnosis to the last follow up or death, respectively. SUV_max_, MTV and TLG were tested in a univariate logistic regression analysis, only SUV_max_ demonstrated to be significantly associated to PRS and OS (*p* = 0.005 and *p* = 0.024 respectively). Multivariate analysis confirmed the results. We found a cut-off of SUV_max_ of 13 that defined worse or better survival (*p* = 0.003). In the first relapse of EOC, SUV_max_ is correlated to PRS and OS, and when SUV_max_ is greater than 13, it is an unfavorable prognostic factor.

## 1. Introduction

Epithelial ovarian cancers (EOC) are the most lethal and silent gynecological tumors with a diagnosis in advanced stages (III–IV) in about 62% of cases [1,2]. The standard approach for treating EOC is surgery and chemotherapy [3,4,5,6,7]. Despite optimal surgery and appropriate first-line chemotherapy, about 70–80% of patients with EOC will develop disease recurrence. Recurrence occurs in 23% of patients during or within 6 months after end of primary chemotherapy, and 60% after six months [8]. Ultrasound, contrast-enhanced tomography (CT), fluorine-18-2-fluoro-2-deoxy-d-glucose PET/CT (^18^F-FDG-PET/CT) and the periodic evaluation of CA 125 levels are the most used methods during the follow up to detect cancer recurrence, even if the correct modalities of follow up are not well defined [9]. A leading option for the treatment of recurrent ovarian cancer is chemotherapy, however in selected cases, resection of the tumor may be considered [10]. The role of surgery in recurrence of EOC is still debated, surgery represents a good option when an absent residual disease (CC0) is present, as demonstrated by the Arbeitsgemeinschaft Gynaekologische Onkologie (AGO) Group DESKTOP OVAR I trial (DESKTOP I) [11], and more recently by the preliminary data of the DESKTOP III from the last ASCO meeting. Data demonstrated a benefit of secondary cytoreductive surgery and chemotherapy, as opposed to chemotherapy alone exclusively in patients with complete resection with a progression-free survival of 5–6 months [12,13]. Good predictive factors of CC0 were macroscopically complete resection at first surgery, good performance status, and the absence of ascites greater than 500 mL. The role of Imaging has become increasingly important, allowing to properly monitor patients, distinguishing the different relapse patterns, thus guiding correct management and therapy. If compared with CT, ^18^F-FDG PET/CT is able to identify recurrence earlier because, in most cases of recurrent, the tissue is characterized by a high consumption of glucose, and therefore in an increased uptake of ^18^F-fluoro-2-deoxyglucose [14]. Recently, the prognostic role of ^18^F-FDG PET/CT through its metabolic parameters has been studied and PET imaging techniques could be used to explore the biological behaviour of tumors during therapy, however there is no consensus on their use [15]. In cervical cancer, our group found that the assessment of the response to therapy based on ^18^F-FDG PET/CT predicts survival in patients with locally advanced cervical cancer treated with concomitant chemo-radiotherapy [16]. In ovarian cancer, some studies proposed that ^18^F-FDG-PET/CT is useful for defining treatment response (Positron Emission Tomography Response Criteria in Solid Tumors–PERCIST criteria) [17,18] to neoadjuvant chemotherapy (NACT) and the method could be a potential predictor of prognosis in NACT and relapse [19]. Based on these premises, the role of ^18^F-FDG-PET/CT as a biological parameter of a tumour to predict prognosis appears promising. The aim of the study is to test the prognostic value of the ^18^F-FDG PET/CT parameters (SUV_max_, MTV and TLG) as prognostic factors in patients with first EOC recurrence.

## 2. Materials and Methods 

### 2.1. Population and Protocol

This is a retrospective study. The clinical data of all patients referred to the Ovarian Cancer Center of Bologna, Italy, from January 2008 to May 2016 were analysed. Among these, we selected patients at first relapse who underwent surgery before chemotherapy. Inclusion criteria were: a) histologically confirmed diagnosis of EOC according to the WHO criteria [20]; b) standard first-line treatment based on cytoreductive surgery and combined platinum-based chemotherapy (carboplatin and paclitaxel 6–9 cycles) c) diagnosis of recurrent EOC confirmed by ^18^F-FDG PET/CT available and performed at our Institute; d) secondary surgery performed in our institution, and e) adequate follow-up over 12 months. The exclusion criteria were: a) borderline and non-EOC; b) patients not evaluated with ^18^F-FDG PET/CT at the time of the first relapse, and c) patients with inadequate information about primary treatment and secondary surgery.

All clinical and pathological data were recovered and examined, including age, body mass index (BMI), histological subtype divided in type I and type II [21], International Federation of Gynecology and Obstetrics (FIGO) stage [22], serum CA 125 levels at the first diagnosis and relapse, chemotherapy schedules and number, surgical information including score of surgical complexity measured with the Aletti’s score [23] and residual disease was divided in the absence of (CC-0) 0.1–0.5 cm, (CC-1) 0.6–1.0 cm, (CC-2) >1 cm, and (CC3) [24]. Periodic clinical and radiological control data were recovered. In our institute, follow-up was performed as follows: CA 125 examination and assessment every four months for the first two years and then every 6 months for five years, CT scan every six months. ^18^F-FDG PET/CT was prescribed whenever there was a clinical suspicion of relapse or as confirmation of another instrumental examination such as CT. According to inclusion criteria, patients with disease relapse are submitted to surgery in case of platinum sensible disease (>12 months to the last chemotherapy) [25,26], single or multiple recurrence amenable to complete surgical removal, the absence of extra-abdominal metastasis, a low level or absence of ascites, low levels of CA125 (≤500 U/mL), and if they were fit for surgery. Otherwise, patients were submitted for chemotherapy without surgery. Progression free survival (PFS) was calculated from the first diagnosis to recurrence, post-relapse survival (PRS) and overall survival (OS) was obtained from the first relapse and from the first diagnosis to the last follow up or death. The study is a part of a larger study that was approved by Comitato Etico indipendente Ospedaliero Universitaria di Bologna on 11th November 2011 (EC number 107/2011/U/Tess). Consent to analyse the data was obtained from the local ethics committee, and informed consent forms were signed by patients and collected. 

### 2.2. Radiopharmaceuticals, Imaging Protocol and Images Analysis

Whole-body ^18^F-FDG PET/CT scans were carried out following standard procedures. Following a 6-h fast, 3 MBq/kg of ^18^F-FDG was intravenously injected in patients. The uptake time was 60 min in all patients on a 3D tomography (Discovery STE; GE) for 2 min per bed position. Cross-calibration was performed using an image quality NEMA phantom. A low-dose CT scan (120 kV, 80 mA) was performed both for attenuation correction and as an anatomical map. PET/CT scans were evaluated by two nuclear medicine physicians experienced in oncology reviewing transverse, coronal and sagittal planes. For each scan, maximum and mean standardized uptake values (SUV_max_ and SUV_mean_), metabolic tumor volume (MTV) and total lesion glycolysis (TLG) were measured. MTV measurement was calculated on PET/CT images using a semi-quantitative analysis (40% threshold). SUV_max_ and SUV_mean_ normalized to body weight were measured within the MTV defined as above. TLG values were calculated as the product of MTV and SUV_mean_ [27]. For each scan, the number of 18F-FDG avid lesions was also measured.

### 2.3. Statistical Analysis

Statistical analysis was performed using SPSS version 24 (IBM Corp., Armonk, NY). The association between the PET parameters (SUV_max_, MTV and TLG), the PRS and OS were investigated by performing a univariate and multivariate analysis (Cox proportional hazard model). An ROC analysis was performed on those PET parameters showing an association with OS in order to carry out a cut-off value useful to predict the risk of mortality. Thereafter, patients were divided into two categories using the cut-off value suggested by ROC analysis. A Kaplan-Meier analysis was performed to show possible different overall survival between these two groups.

## 3. Results

### 3.1. Population and Clinical Data

**Figure 1:**
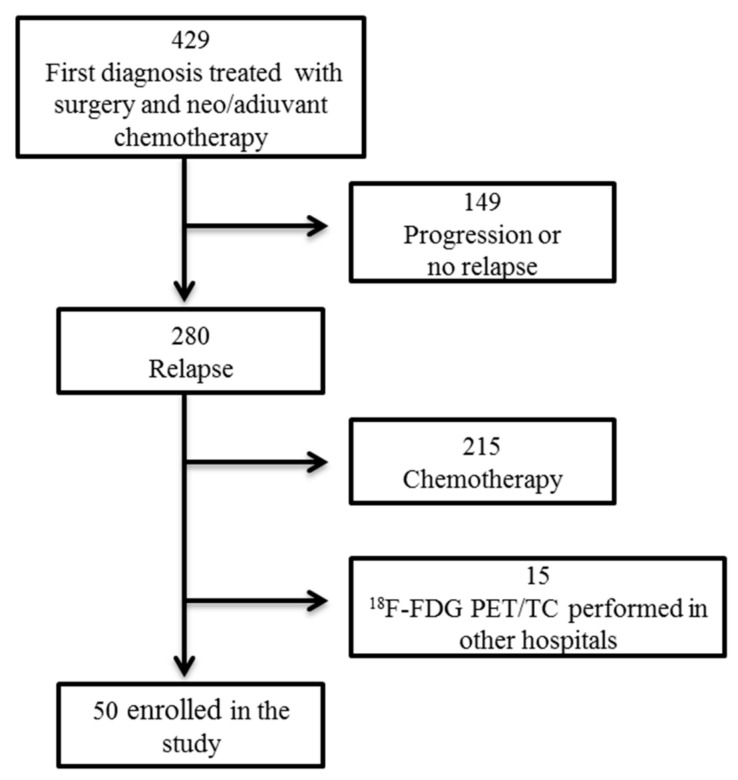
Flow chart of the study. Patient’s selection from our database of patients with ovarian cancer.

The flow cart of the recruitment is shown in Figure 1. Characteristics of the 50 patients are reported in Table 1; the majority of patients present type II (82%) diseases and were in an advanced stage (78%) with about 34% of patients undergoing neoadjuvant therapy before surgery. The suspicion of recurrence was represented by clinical symptoms (intestinal discomfort and abdominal pain) in 4/50 (8%), increased blood levels of CA 125 in 14/50 (28%), ultrasound 4/50 (8%), CT scan 24/50 (28%) and ^18^F-FDG PET/CT 4/50 (8%). Recurrence occurred after a disease-free survival (DSF) of 36.3 ± 40.13 months (mean ± SD—standard deviation) and the levels of CA 125 were significantly lower (*p* = 0.001) in the relapse than in the first diagnosis. 

### 3.2. Surgical Data 

At first diagnosis, optimal residual disease (CC-0) was achieved in 90% of cases. Surgical complexity was significantly lower (*p* = 0.001) in the relapse than in primary surgery. Six patients (12%) who underwent surgery were judged not optimal cytoreducible for disease extension; 44 patients (88%) received optimal debulking surgery (CC0). The Aletti’s score in secondary surgery was lower in relapse than primary surgery (*p* = 0.009) (Table 1). 

### 3.3. Follow Up Data

**Figure 2:**
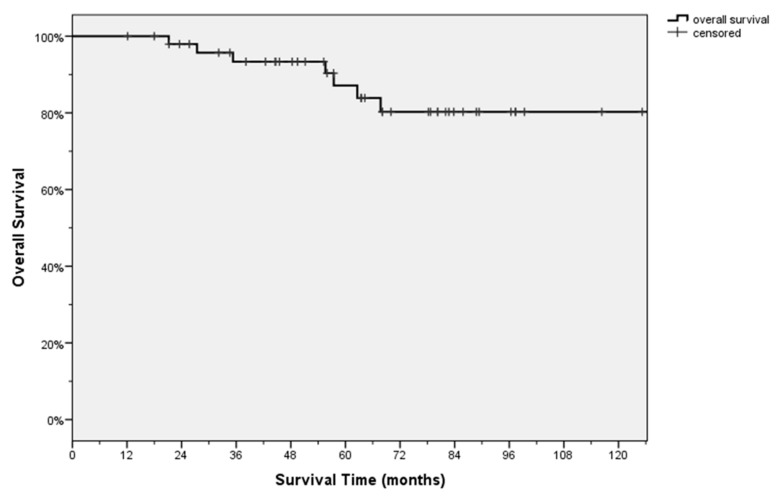
Kaplan-Mayer-Analysis of Overall Survival (OS) of the 50 patients enrolled in the study.

During the follow-up period, 6 patients died after 44.1 ± 18.7 months (mean ± SD) and 13 relapsed after 21 ± 7.7 (mean ± SD) months and received subsequent lines of chemotherapy. The mean follow-up was 70.2 ± 48.3 months, the 5-year OS was 87% (Figure 2).

### 3.4. PET’s Data Analysis 

According to PET data analysis, ^18^F-FDG PET/CT showed a single positive lesion in 19/50 (38%) of cases, multifocal disease in 23/50 (46%) and diffuse (carcinomatosis) in 8/50 (16%). The average number of lesions identified by PET was 3.4 ± 3.6 (mean ± SD) (range 1–6). The correspondence between ^18^F-FDG-PET/TC and surgical evaluation was observed in 94% of cases. The SUV_max_, MTV and TLG values were 11 ± 5.6, 33.4 ± 10.5, 246.1 ± 946.7 (mean ± SD), respectively.

The univariate Cox analysis showed a correlation between SUV_max_ values and PRS (*p* = 0.005) with an odds ratio (OR) = 1,244 (95% CI = 1068–1447) and OS (*p* = 0.024) with an odds ratio (OR) = 1177 (95% CI = 1021–1356). No correlation was observed between MTV and TLG with PRS (*p* = 0.316 and *p* = 0.074, respectively) and OS (respectively *p* = 0.162 and *p* = 0.106).

**Figure 3:**
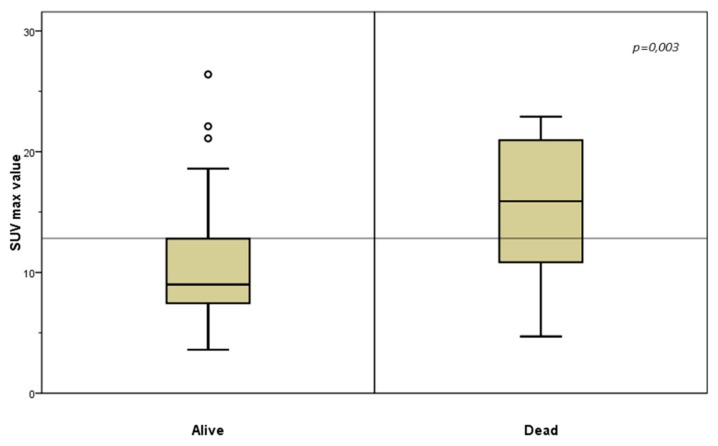
**Standardized Uptake Values** (SUV_max_) value and Overall Survival. SUV_max_ greater than 13 represents a poor prognostic factor.

The multivariate Cox analysis was performed by testing the following variables SUVmax, TLG and MTV with the Wald backward method (Table 2). The analysis showed that the best model predicting the OS was the SUV_max_ variable alone. The ROC analysis showed that the best cut-off for SUV_max_, in this cohort of patients, was 13 (Figure 3). 

**Figure 4:**
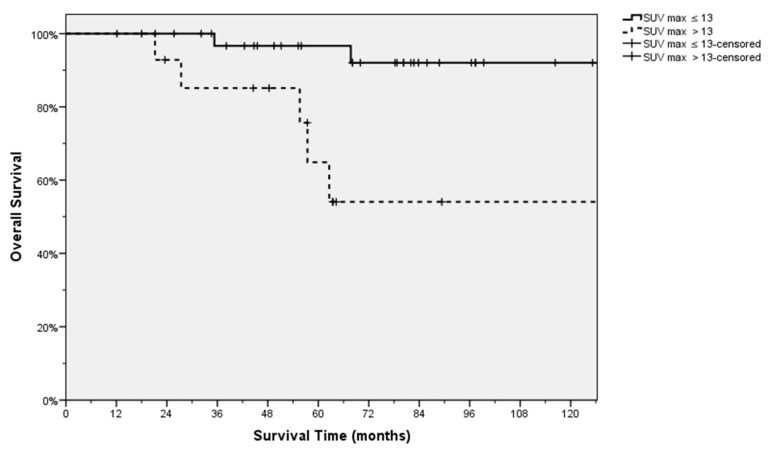
Different Overall Survival (OS) of the patients divided on the basis of the SUVmax value.

Therefore, patients were divided into two groups by using this value. A Kaplan-Meier performed between these two groups showed patients with a SUV_max_ value lower than or equal to 13 had a significantly better OS (*p* = 0.003) (Figure 4). 

Moreover, it was investigated the association between SUV_max_ and CA 125 values at relapse by using the Pearson correlation test; no statistical correlation was found between these two variables (*p* = 0.264).

## 4. Discussion

This pilot study, performed in a selected population of EOC relapse patients, lays out the clinical foundation to investigate the PET parameters, such as SUV_max_, MTV and TLG as prognostic factors in addition to the existing ones during EOC relapse. Particularly, we found that one of these parameters, SUV_max,_ is correlated with PRS and OS. To the best of our knowledge, this is the second study of its kind to explore the possible prognostic role of ^18^F-FDG PET/TC in EOC relapse.

Although our series is a selected group of patients, it can be representative of a larger group of relapsing patients undergoing surgery, taking into account some parameters: the high incidence of recurrence found in the initial population (80%) and the high number of patients selected for chemotherapy (215 patients) compared to those undergoing surgery (65 patients) as described in the literature [10,11,13,28]. Our data showed a good selection of patients suitable for surgery, as evidenced by the high percentage of CC0 (82%) and the high overall survival of the population at 5 years (87%). We chose to study patients submitted to surgical procedures because surgery represents the gold standard to confirm the diagnosis of relapse, and to compare the characteristics found with PET. In our study, we observed a good agreement between the two assessments in 94% of cases. Moreover, ^18^F-FDG-PET/TC combines the best features of PET with CT and has been shown to have a sensibility and specificity of 91% and 88%, respectively, and a predictive positive value (PPV) of 94%. PET/CT in EOC relapse is more accurate than other imaging methods in detecting small carcinomatosis implants, lymph node involvement, as well as chest and bone metastasis [29].

The literature data demonstrate that when EOC recurs, it should be considered a chronic and lethal disease with poor prognosis [11]. In these cases, different therapeutic options should be proposed, in particular clinical studies and new therapeutic strategies that should be different from case to case basis. The well-known intertumoral and intratumoral heterogeneity of ovarian cancer excludes the likelihood of finding a single therapy that can be curative for most patients, and therefore requires the development of tools that can instead lead to individualized therapy [30].

At the time of the first relapse, recent studies have reported that the surgical approach with no residual disease (CC0) associated with chemotherapy has led to a better prognosis than chemotherapy alone [31]. To obtain these results, it is important to select patients who will initially benefit from surgical treatment. The DESKTOP studies [10,11,13] have tried to define the profile of suitable candidates for surgery, taking into account the patient’s performance status, biological tumour aggressiveness (from stage and residual tumour to first diagnosis) and actual diffusion of the disease (presence of ascites). None of these parameters, however, consider the intratumoral and intertumoral heterogeneity of recurrence, which is a “hot spot” in ovarian cancer therapy. In our study, we selected patients for surgical treatment according to the current guidelines, but also attempted to understand tumour biodiversity using PET parameters.

Despite the potential ability of ^18^F-FDG PET to study tumour metabolism in vivo, this issue is poorly investigated. The possible role of PET’s parameters based on volume and uptake intensity, such as SUV_max_, MTV, TLG and their possible impact in predicting tumour biology, should be explored with different intentions: to monitor therapy response, study heterogeneity of the tumour and for the early identification of patients who are candidates for surgery or chemotherapy [32]. ^18^F-FDG is a glucose analogue that is preferentially taken up by metabolically active cells (normal and neoplastic cells). Neoplastic cells tend to show high levels of uptake, due to their greater dependence on glucose [33]. Aggressive tumours, and in particular their metastases, increase glycolysis and suppress oxidative phosphorylation, suggesting that an increase in glycolysis preference may be a hallmark of the metastatic and aggressive phenotype. A high glucose uptake could be compatible with an aggressive tumour as a sign of a glycolytic tumour and this can probably be exploited for the imaging of metabolically active tumours using ^18^F-FDG PET/CT [15,16]. Based on these assumptions, we tried to correlate ^18^F-FDG PET/CT metabolic parameters to OS and we found that SUV_max_ represents a prognostic factor (*p* = 0.024) of aggressiveness and the cut-off 13 represents a marker of poor prognosis (Figure 3). No correlations between prognosis and TGL and MTV were found. Our data are supported by a follow up longer than five years (Figure 2).

In a previous retrospective study, Kim et al. [19] evaluated the prognostic value of quantitative metabolic parameters of ^18^F-FDG-PET/CT at the time of the first relapse in patients with EOC relapse. Results of this study showed that quantitative metabolic parameters measured with ^18^F-FDG-PET/CT at the time of first relapse were significant predictors of prognosis. Univariate and multivariate analyses demonstrated that whole-body metabolic tumor volume and whole-body total lesion glycolysis were independent predictors of prognosis. However, SUV_max,_ analyzed as continuous variable, had no correlation with prognosis, however the same authors found that a cut-off higher than 14 in the SUV_max_ defines a worse course of the disease. 

In the literature, prognostic factors and predictive response to therapy of the PET parameters were explored in several tumours and the most extensive studies have been performed on the lung and o oesophagus with conflicting results. With regard to lung cancer, 21 retrospective studies, including 2637 patients with stages I to IV non squamous cellular lung cancer (NSCLC), found that a high SUV_max_ was associated with poor prognosis [34], and a second meta-analysis, limited to patients with stage I NSCLC, found that a lower FDG uptake was associated with a better prognosis [35,36]. In a meta-analysis of seven studies in oesophageal cancer that evaluated the impact of SUV_max_ on overall survival, a high SUV predicted a worse survival [37], but data were not confirmed in a large retrospective series [38]. The results suggested a better response to preoperative chemoradiotherapy in the group with high SUV_max_. 

The main limitations of the study included the small number of patients enrolled and the retrospective analysis which could constitute a bias; data should be confirmed in a larger and prospective series of patients, probably including EOC relapse in chemo-sensible and chemo-insensible patients. 

## 5. Conclusions

In conclusion, ^18^F-FDG PET/CT is a diagnostic method that combines anatomical imaging with molecular behaviour of cancer cells. The uptake of ^18^F-FDG reveals the heterogeneity of tumours and if associated to clinical, surgical and pathological parameters, could contribute to the development of a therapeutic choice tailored on a patient-by-patient basis. Indeed, ^18^F-FDG PET/CT may represent an alternative approach to characterize relapsed ovarian tumours. In the future, clinicians should consider the metabolic information provided by ^18^F-FDG PET/CT in the therapeutic choices for their patients.

## Figures and Tables

**Table 1:** Clinical and surgical parameters in our patients at first diagnosis and relapse.

	First Diagnosis	Relapse	*p*
**Age**			
(Mean ± SD)	53.0 ± 9.2	55.7 ± 9.5	*ns*
**Body mass Index (BMI)**			
(Mean ± SD)	24.2 ± 6.7	25 ± 5.6	ns
**Histological parameters**			
Type 1	9 (18%)		
Type 2	41 (82%)		
Serous	35 (70%)		
Mucinous	1 (2%)		
Endometrioid	11 (22%)		
Clear cell	3 (6%)		
**Tumor Grading**			
G1	2 (4%)		
G2	6 (12%)		
G3	42 (84%)		
**FIGO stage**			
I	5 (10%)		
II	5 (10%)		
III	37 (74%)		
IV	3 (6%)		
**Genetic mutations**			
BRCA 1	4 (8%)		
BRCA 2	2 (4%)		
Missmatch repair (MMR)	1 (2%)		
No mutations	43 (86%)		
**Bevacizumab**			
Yes	6 (12%)		
No	44 (88%)		
**SUVmax**			
(Mean ± SD)		11 ± 5.6	
**TLG**			
(Mean ± SD)		250.9 ± 946	
**MTV**			
(Mean ± SD)		34 ± 105.63	
CA 125 (U/mL)			
0–34	3 (6%)	20 (40%)	
35–499	18 (36%)	25 (50%)	
500–999	9 (18%)	1 (2%)	
≥1000	14 (28%)	2 (4%)	
not available	6 (12%)	2 (4%)	*0.001*
**ALETTI SCORE**			
Low complexity	13 (26%)	27 (54%)	
Mediun complexity	26 (52%)	19 (38%)	
High complexity	11 (22%)	4 (8%)	*0.001*
**RESIDUAL DISEASE**			
CC0	45 (90%)	44 (88%)	
CC1	4 (8%)	0 (0%)	
CC2	1 (2%)	2 (4%)	
CC3	0 (0%)	4 (8%)	*ns*
**Time between first relapse and death**			
(Mean ± SD)		27.8 ±14.3	

Legend: FIGO stage: International Federation of Gynecology and Obstetics, SUVmax: maximum standardized uptake values, MTV: metabolic tumor volume, TGL: total lesion glycolysis, SD: standard deviation.

**Table 2:** Associations with PET to Overall Survival.

PET Parameters		*p*-value	Odds Ratio	95% CI
Step 1	Standardized Uptake Values (SUVmax)	0.257	1.103	0.931–1.306
	Metabolic Tumor Volume MTV	0.273	0.928	0.812–1.060
	Total Lesion Glycolysis (TLG)	0.180	1.010	0.996–1.024
Step 2	Standardized Uptake Values (SUVmax)	0.201	1.125	0.939–1.347
	Total Lesion Glycolysis (TLG)	0.446	1.002	0.997–1.006
Step 3	Standardized Uptake Values (SUVmax)	0.024	1.177	1.021–1.356
